# Draft Genome Sequence of a Multidrug-Resistant *Serratia marcescens* Strain, Isolated from a Patient with Peritoneal Cancer in South Africa

**DOI:** 10.1128/genomeA.00580-17

**Published:** 2017-06-29

**Authors:** Ashika Singh-Moodley, Olga Perovic, Senzo Mtshali, Arshad Ismail, Mushal Allam

**Affiliations:** a Centre for Healthcare-associated infections, Antimicrobial Resistance and Mycoses, National Institute for Communicable Diseases, National Health Laboratory Service, Johannesburg, South Africa; b University of Witwatersrand, Johannesburg, South Africa; c Sequencing Core Facility, National Institute for Communicable Diseases, National Health Laboratory Service, Johannesburg, South Africa

## Abstract

We report here the draft genome sequence of *Serratia marcescens* ML2637, isolated from a South African pediatric patient in the intensive care unit with peritoneal cancer. The genome comprised 5,718,350 bp, with a 59.1% G+C content. There were 5,594 predicted genes, including 5,301 protein-coding genes, 199 pseudogenes, and 94 RNA genes.

## GENOME ANNOUNCEMENT

*Serratia marcescens* is a motile, short, rod-shaped, Gram-negative, facultative anaerobic bacterium, classified in the large family *Enterobacteriaceae*. An Italian pharmacist discovered it on spoiled polenta in 1819 (1). Since then, *S. marcescens* is often found in the soil, water, and animals, as well as on plants (2, 3). Along with other opportunistic and nosocomial pathogens, *S. marcescens* is involved in hospital-acquired infections, particularly catheter-associated bacteremia and urinary tract and wound infections (4, 5). Here, we present the draft genome sequence of *S. marcescens* ML2637, recovered from a blood culture isolated from a 9-year-old patient with peritoneal cancer in the intensive care unit of an academic hospital in South Africa.

We sequenced the isolate because antimicrobial susceptibility testing showed that the isolate was phenotypically resistant to multiple classes of antibiotics. We therefore required confirmation with genotypic resistance.

Genomic DNA was extracted using the QIAamp DNA mini kit (QIAGEN, Germany). Paired-end libraries (2 × 300 bp) were prepared using the Nextera XT DNA sample preparation kit (Illumina, USA). Sequencing was performed on an Illumina MiSeq machine. The resulting paired-end reads (2,969,552 reads) were merged, quality trimmed, and *de novo* assembled using the Qiagen CLC Genomics Workbench version 10 (Qiagen, Netherlands). The assembly contains 224 contig sequences longer than 500 bp, comprising 5,718,350 bp with a G+C content of 59.1% and an *N*_50_ of 62,301 bp. All contigs were then submitted to GenBank, where gene annotation was implemented using the NCBI Prokaryotic Genome Annotation Pipeline (PGAP) (6). Among the 5,594 genes predicted by PGAP, 5,301 were protein-coding genes, 199 were pseudogenes, and 94 were RNAs (5 rRNAs [5S, 16S, and 23S], 74 tRNAs, and 15 ncRNAs). The annotation was further uploaded to the Rapid Annotations using Subsystems Technology (RAST) server for subsystems-based annotation (7–9). The RAST annotation assigned 4,117 genes (73.59%) into 591 subsystems, with the maximum number of genes associated with carbohydrates (14.84%), followed by metabolism of amino acids and derivatives (12.43%) and membrane transport (7.91%). By using ResFinder (10), several antibiotic resistance genes were found in this genome, including the beta-lactamase resistance genes *bla*_SRT-2_ and *bla*_NDM-1_, the tetracycline resistance genes *tet*(A) and *tet*(41), the aminoglycoside resistance genes *rmtC* and *aac*(6′)-Ic, the sulfonamide resistance gene (*sul1*), and the trimethoprim resistance gene (*dfrA14*).

### Accession number(s).

This draft genome sequence has been deposited at NCBI GenBank under the accession number NDXU01000000.
